# Legacy for Young Men: PFAAs and Human Sperm

**DOI:** 10.1289/ehp.117-a256b

**Published:** 2009-06

**Authors:** Cynthia Washam

Recent studies suggest that men’s capacity for sperm production may be harmed by toxic exposures in both fetal and later life. Among the potential chemical culprits are the perfluoroalkyl acids (PFAAs), highly persistent degradation products of the polyfluorinated compounds used in products including nonstick cookware and water-resistant coatings for carpeting, clothing, and other textiles. Findings from a new Danish study suggest that exposure to PFAAs may help account for the otherwise unexplained poor semen quality observed in many young men today **[*EHP*** **117:923–927; Joensen et al.]**.

Studies in the 1990s found that PFAAs diminished testosterone levels and increased estradiol levels in male rats. In human studies, men have appeared to have higher serum PFAA concentrations than have even higher levels compared with those of older men. Young men may therefore be at higher risk for any potential adverse effects posed by these chemicals.

Inspired by such observations, the Danish team designed what they believe is the first study of the effects of PFAAs on sperm quality in humans. They focused their investigation on perfluorooctanoic acid (PFOA) and perfluorooctane sulfonic acid (PFOS) because of these compounds’ prevalence, their long half-lives, and existing evidence that they act as endocrine disruptors. The subjects included 105 young men from the general population who had provided semen samples in 2003 as part of Denmark’s compulsory military draft examination. These men had the highest and lowest testosterone counts of 546 potential subjects considered. Serum levels of PFOA and PFOS were combined to give each man a PFAA score. These scores were used to classify subjects into low-, intermediate-, and high-PFAA groups.

Sperm quality varied markedly among all three groups. Compared with the low- and intermediate-PFAA groups, men in the high-PFAA group showed significantly poorer sperm quality in terms of both percentage and total numbers of morphologically normal sperm. Ejaculate from men in the low-PFAA group had a median count of 15.5 million normal sperm compared with 10 million and 6.2 million normal sperm in the intermediate- and high-PFAA groups, respectively. Average sperm concentration and motility also were lower in the high-PFAA group, but not significantly so. PFAAs were not inversely associated with testosterone levels, a finding that was contrary to expectations based on previous animal research.

The researchers note that humans and wildlife will be exposed to persistent PFAAs for years to come. They speculate that high exposures to PFAAs may be contributing to low semen quality and subfertility reported in other studies. However, they caution, results from this preliminary study should be corroborated in larger studies.

## Figures and Tables

**Figure:**
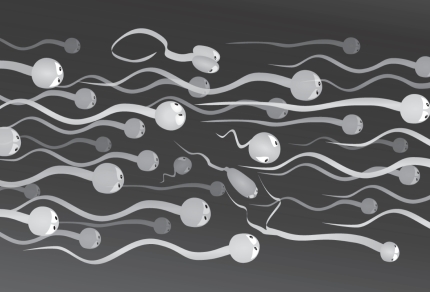
Men with the highest PFAA scores also had the highest percentages and numbers of morphologically abnormal sperm.

